# Physician Suitability of an EHR Interface for Depicting Ecological Symptoms Derived From a Smartwatch

**DOI:** 10.1093/geroni/igaa057.2896

**Published:** 2020-12-16

**Authors:** Todd Manini, Jordan Alpert, Tonatiuh Mendoza, Satya Prabhakar, Laurence Solberg, Parisa Rashidi

**Affiliations:** 1 The University of Florida, Gainesville, Florida, United States; 2 University of Florida, gainesville, Florida, United States; 3 University of Florida, Gainesville, Florida, United States; 4 ANSYS Inc, Canonsburg, Pennsylvania, United States

## Abstract

Physicians desire an electronic health record (EHR) interface to visualize data from mobile devices in rapid and unobtrusive manner. We developed an EHR interface after performing semi-structured qualitative interviews with 12 physicians. These interviews led to creating an EHR graphical interface that allowed seamless viewing of the top ranked patient attributes of pain, falls, hydration and mobility patterns. Physicians then evaluated the interface using the International Standard ISO 9241, Part 110— a survey to evaluate the design of human computer interactions. The median response for each of domains were as follows: suitability = 18.5 out of 24 (range: 9-24); conformity=34 out of 40 (range: 16-40); self-descriptiveness=25 out of 32 (range: 13-31); controllability=19.5 out of 24 (range: 8-23); error tolerance=5 out of 8 (range: 1-8). The median total score of 111.5 out of 140 (range: 88-128) indicated a relatively high level of usability of the EHR interface depicting smartwatch data.

